# Supplementary material for: New gene annotations for three pea aphid genome assemblies allow comparative analyses of genes and gene family evolution
Source: bioRxiv. 2025 May 13:2025.05.08.652899. Preprint. [Version 1] doi: 10.1101/2025.05.08.652899 (PMC12132395; doi:10.1101/2025.05.08.652899)
Supplement: Supplement 1 [file media-1.zip › Supp_Mat_for_Jenn/Supplemental_Figures.pdf]

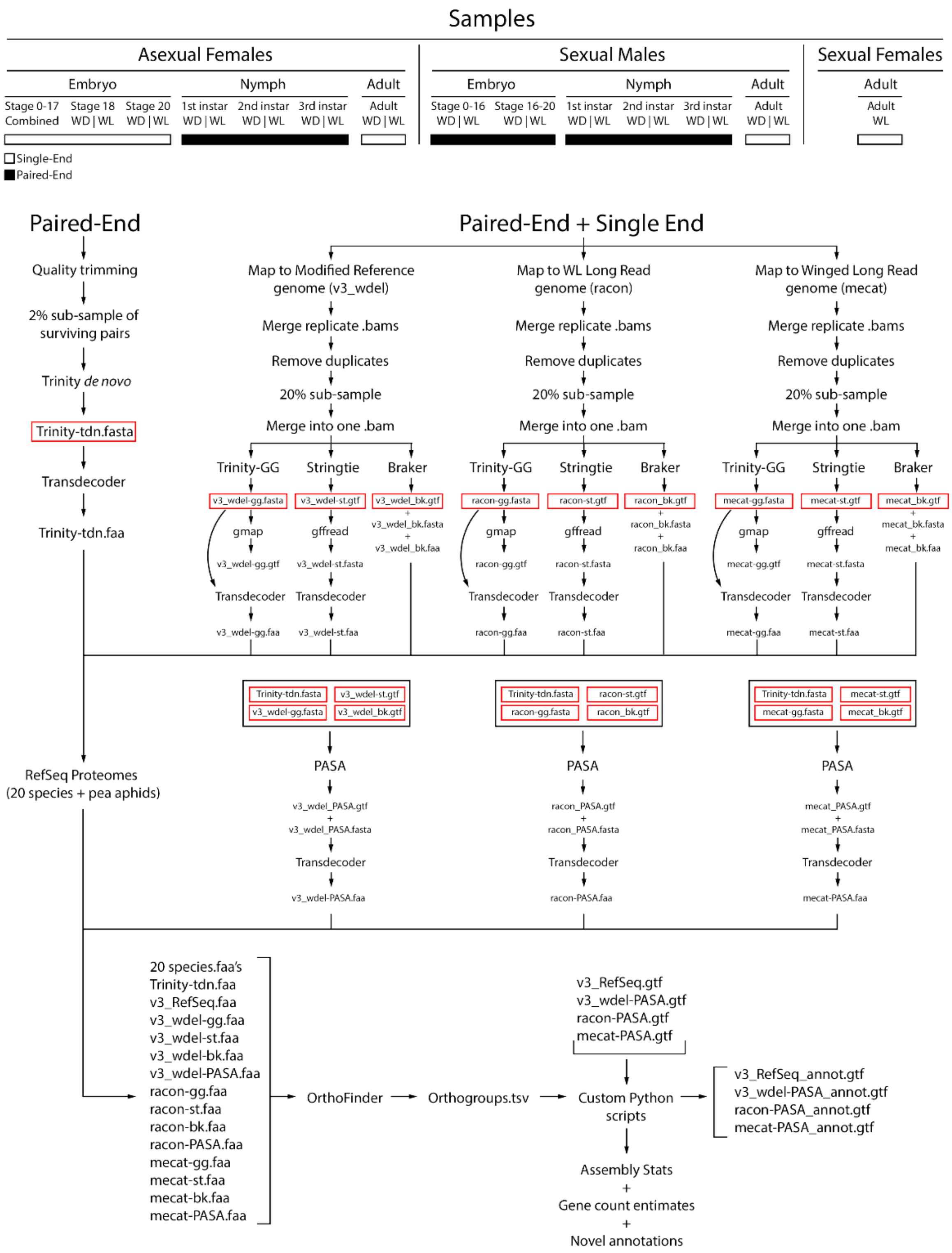

Supplemental Figure S1. Workflow diagram of genome annotation.

Information regarding RNAseq samples used in this study are shown at the top with Single-End read samples indicated by a white bar and Paired-End read samples indicated by a black bar. Transcript files from four methods for each assembly used as input to PASA are boxed in red.
